# Gut–testis axis: how microbiota influence male reproductive health

**DOI:** 10.2478/abm-2026-0017

**Published:** 2026-06-30

**Authors:** Ruiyang Hu, Jingxing Wu, Nianben Chen, Wenjun Ma, Qinglin Hu

**Affiliations:** Department of Clinical Medicine, The Second Clinical Medical College of Anhui Medical University, Hefei 230032, Anhui, China; The First Clinical Medical College of Anhui University of Chinese Medicine, Hefei 230022, Anhui, China; Department of Urology, Chuzhou Integrated Hospital of Chinese and Western Medicine, Affiliated to Anhui University of Traditional Chinese Medicine, Chuzhou, China

**Keywords:** gastrointestinal microbiome, gut dysbiosis, gut–testis axis, male reproductive health, men’s health, microbiota, testis

## Abstract

The intestinal flora forms a complex ecosystem that interacts with the host, influencing health and fitness through mechanisms that connect with distant organs like the brain, liver, muscles, and testes. The gut microbiota plays a vital role in regulating androgen production and metabolism, and can cross the blood–testis barrier to influence spermatogenesis. This review highlights the significance of the gut–testis axis in male reproductive and sexual health, based on extensive studies exploring how gut microbes impact testicular function. Gaining this understanding deepens our knowledge of the gut–testis axis and its role in male reproductive health.

Trillions of beneficial microorganisms, including bacteria, fungi, and viruses, reside in the human body and are vital for health [1]. Among these, the intestinal flora is particularly significant, containing about 10 times more cells and 150 times more deoxyribonucleic acid (DNA) than the host. It weighs approximately 1.5 kg, nearly equivalent to the liver [2, 3]. As a result, the gut microbiota is regarded as a vital organ within the human body “superorganism” [4]. The connection between gut microbial dysbiosis and human diseases has been widely recognized, including conditions like inflammatory bowel disease [5, 6].

The gut microbiota interacts not only locally within the gut but also through a network involving various distant organs and tissues. Its essential role in multiple systemic interactions has been confirmed, including the gut–brain axis [7], the gut–liver axis [8], the gut–muscle axis [9], and the gut–kidney axis [10]. Recently, the influence of the gut microbiota on the male reproductive system has garnered significant attention, with research on the gut–testis axis increasing annually. The gut microbiome is now considered a potential biomarker for male hypogonadism, as studies have found links between gut microbial profiles and testosterone levels [11]. Consequently, research has been proposed to develop new treatments for testicular dysfunction through modification of the gut microbiome [12]. Additionally, gut microbiota transplantation has been shown to improve semen quality [13].

In this review, we conduct a thorough examination of the existing literature on communication between the testis and the gut microbiota, as well as their connection to male reproductive issues caused by gut microbial imbalances. Due to the study’s complexity, we primarily focus on the role of the gut–testis axis, but also acknowledge that other signaling pathways may be involved. Additionally, we highlight the opportunities and challenges that future research in this area presents. Overall, the gut–testis axis illustrates how intestinal microorganisms and their metabolites impact androgen production, metabolism, and spermatogenesis, thereby supporting overall reproductive function as shown in **Figure 1**.

**Figure 1.**
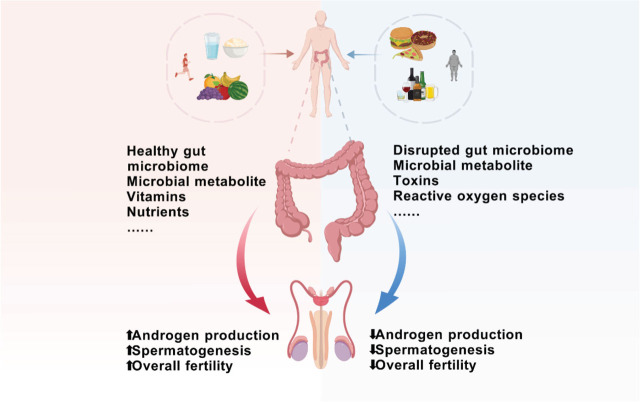
Influence of intestinal microorganisms on testicular function.

## Influence of the gut–testis axis on testicular function

The gut–testis axis is a complex bidirectional communication system where alterations in gut microbiota can lead to systemic changes and inflammatory responses that negatively affect the testicular environment and sex hormone levels [14]. Androgens, key hormones produced in the male testes, can influence gut microbiome composition through intricate mechanisms. Maintaining a healthy lifestyle, including a balanced diet, probiotics, and botanicals, can promote a balanced gut microbiome, supporting male reproductive health [15]. Conversely, substances such as toxins, endocrine disruptors, and heavy metals can disturb intestinal stability and harm reproductive health and hormone balance. Gut microbes can modulate androgen levels by regulating androgen metabolism, the study highlighted significant differences in the levels of glucuronidated androgens and free androgens in the intestinal contents of the small intestine and more distal regions of the intestine, depending on the gut microbiota [16].

**Figure 2.**
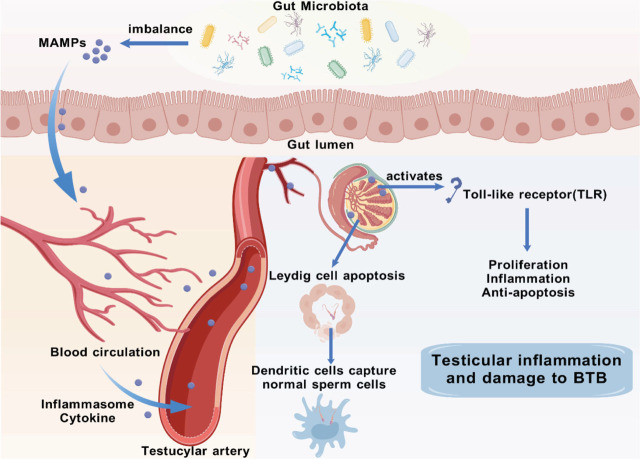
Gut microbiota imbalance leads to testicular inflammation and BTB damage. BTB, blood–testis barrier; TLR, toll-like receptor.

The blood–testis barrier (BTB) is formed by cell junctions between supporting cells at the base of the seminiferous tubules. It includes various types of junctions, such as tight junctions, gap junctions, and other cell-like junctions, which are made up of capillary endothelium, basement membrane, connective tissue, and supporting cells [17]. These junctions mainly exhibit basic electrophysiological features. Regulatory factors in cell–cell junctions of the testis include hormones, cytokines, growth factors, nitric oxide (NO), and the gut microbiota. Dysbiosis of the gut microbiota can lead to an overgrowth of harmful microorganisms, increasing pro-inflammatory markers. This can be attributed to the adverse effects of inflammation and oxidative stress caused by Lipopolysaccharide (LPS), which activate immune cells like dendritic cells and macrophages. These cells enter the testes through the lymphatic system, blood vessels, or other pathways, disrupting the immune environment of sperm and affecting their health. Studies indicate that LPS can cause orchitis and significantly decrease the expression of cell connections [18]. Elevated LPS and systemic endotoxemia trigger inflammation and damage cell connections, impairing the typical structure of the BTB. Disruption of the intestinal barrier allows pathogens and proinflammatory cytokines to enter the bloodstream (**Figure 2**).

### Probiotics

The mechanism of action of probiotics may differ between strains or result from multiple strains and processes. Its main mechanisms include producing antimicrobial enzymes or metabolites, competing for binding sites, inhibiting pathogenic bacteria, competing for nutrients, and modulating the immune system responses [19]. A study involving 52 men with idiopathic oligozoospermia treated with probiotics found that daily supplementation with 500 mg probiotic capsules (including *Lactobacillus casei*, *Lactobacillus rhamnosus*, *Lactobacillus bulgaricus*, *Lactobacillus acidophilus*, *Bifidobacterium breve*, *Bifidobacterium longum*, and *Streptococcus thermophilus*) for 10 weeks significantly improved infertility. The strains *L. acidophilus*, *B. breve*, *B. longum*, and *S. thermophilus* increased sperm concentration and viability in infertile men, and also reduced oxidative stress and inflammatory markers. This is analyzed because probiotics can enhance the body’s antioxidant capacity, increase the neutralization of reactive oxygen species (ROS), and lower ROS levels through the nuclear factor erythroid 2-related factor 2 (Nrf2) pathway genes [20]. Another study involving 56 men with idiopathic infertility treated with FamiLact (a probiotic plus prebiotic) found that taking 500 mg daily of FamiLact (which includes *L. rhamnosus*, *L. casei*, *L. bulgaricus*, *L. acidophilus*, *B. breve*, *B. longum*, *S. thermophilus*, and fructooligosaccharides) for 80 d significantly improved sperm concentration, viability, and morphology, while reducing oxidative stress in semen and thereby decreasing sperm DNA damage [21].

### Nutrient

Gut flora dysbiosis also affects nutrient synthesis and may impair testicular function [22]. Essential nutrients such as vitamins A, K, folic acid, calcium, and others are impacted. Vitamin A is an indispensable nutrient for reproductive and embryonic development, and one of its metabolized forms is retinoic acid, which promotes the differentiation of spermatogonial stem cells into mature spermatids [23]. A diet rich in vitamin K boosts the testicular anti-inflammatory response and raises serum testosterone by influencing cholesterol and steroid hormone genes [24]. Calcium is essential for fertilization as it controls sperm motility and sperm–egg binding. Sperm capacitation depends on calcium channel activation on the sperm tail, which promotes sperm movement into the female reproductive tract [25]. Nutrients produced and processed by intestinal microbes play a vital role in testicular function. Accordingly, we investigated their impact on reproduction, summarizing the key producing bacteria (**Table 1**).

**Table 1.** Summarizes the nutrients produced by gut microbiota that influence the male reproductive system

**Main bacteria**	**Nutrient**	**Function in the male reproductive system**
*Escherichia coli Clostridia* [26]	Vitamins A	Encourages spermatogonial stem cells to differentiate into sperm
*Bacteroides fragilis* [26]	Vitamins K	Resistance against the inflammatory response
		Promotes serum testosterone and the blood–testis barrier
*Lactobacillus Bifidobacterium Acidobacillus* [27]	Folic acid	Encouraging germ cell differentiation
		Anti-inflammatory and antioxidant
*Bifidobacteria Lactobacillus* [28]	Calcium	Enhances sperm motility and capacitation
		Initiates the acrosome reaction Signal transduction in germ cells

### Dietary patterns

Recently, there has been a steady rise in studies examining the link between disease and health issues through dietary patterns, which can offer a more comprehensive view of the complex relationship than studies focusing on individual nutrient intake. Results of a randomized controlled trial showed a significant improvement in sperm motility in obese men after a 16-week low-energy diet (800 kcal/d) combined with a brief dietary intervention, but its impact on fertility outcomes still needs further investigation [29]. Other studies have shown that losing weight through a low-calorie diet (800 kcal/d) for 8 weeks increases sperm concentration and sperm count, and that these levels are maintained after 1 year in men who keep the weight off loss [30]. Studies also show that men with healthy diets have longer sperm telomeres, indicating that sperm quality is closely linked to an individual’s lifestyle and diet habits [31].

### Influence of testicular function on the intestinal–testicular axis

The testes, located outside the body in the scrotum, are exposed to both internal and external changes. Factors such as temperature stress, hormone levels, endocrine disruptors, diet, physical activity, growth, development, and congenital conditions can all impact testicular function [32]. Gamete development involves germ and somatic cells. Mature testes generate spermatozoa, which are stored in the epididymis and expelled through the seminal ducts via the penis during ejaculation. This process depends on nutrients such as water, amino acids, fats, carbohydrates, vitamins, and minerals, as germ cells differentiate and mature by exchanging nutrients and waste with supporting cells [33]. Since the testes cannot synthesize nutrients, blood vessels deliver these from the digestive system to the testicular interstitium through capillaries, which cross into the interstitial tissue via mast cells and junctions. Essential nutrients—such as vitamin A, folic acid, vitamin K, calcium, and others—are crucial for maintaining testicular structure and function.

Androgens influence the gut microbiome by impacting the gut barrier and environment. Research shows that testosterone induces changes in the gut microbiota of male and female mice around puberty, partly due to elevated sex hormone levels, mainly testosterone [34]. Additionally, androgens can affect the proliferation and differentiation of intestinal stem cells by negatively regulating bone morphogenetic protein (BMP) signaling through the androgen receptor on intestinal stromal cells [35]. The study shows that Androgen-deprivation therapy (ADT) usage is associated with increased cardiovascular disease-related mortality risk for patients diagnosed with prostate cancer compared with ADT non-users [36]. In conclusion, androgens may modify gut microbes by altering the gut barrier and environment, but their influence on gut homeostasis can be a double-edged sword.

## Opportunities and challenges

The gut–testis axis is crucial for understanding male reproductive and sexual health [37]. Further research could establish the role of specific bacteria in influencing these health aspects and offer new treatments for conditions like hypogonadism, infertility [38], and sexual dysfunction by restoring a healthy gut microbiota. This opens possibilities for exploring probiotics, microbial agents, and other strategies to manage these issues. It has been observed that taking oral probiotics (including *Lactobacillus*, *Bifidobacterium*, etc.) can reduce the inflammatory and oxidative responses of sperm, thereby improving sperm health quality [20, 39].

Research on whether the gut–testis axis influences male reproductive and sexual health still faces several challenges. First, due to limited studies on the physical characteristics of the male testis in different microbiota states and the gut microbiota in male reproductive disorders, there is an urgent need to clarify how specific gut microbiota relate to reproductive traits. Specifically, understanding what defines “healthy” versus “sick” microbiota and identifying taxa that are harmful or beneficial to testicular health in both animals and humans is crucial. Second, the main challenge is understanding how the gut–testis axis communicates and functions, including how various molecules influence testicular activity. Candidate molecules, such as short-chain fatty acids like butyric acid, endotoxins, ROS, and pro-inflammatory cytokines, are released or triggered by the gut microbiota [40]. It is also possible that circulating testosterone produced by the testes affects the microbiota by altering the gut environment and immune response, which invites further research into the gut–testis axis [41, 42]. Currently, no studies definitively link gut microbial imbalance to impaired testicular function in humans. Although animal models with specific genetics, diets, environments, and microbiomes offer valuable insights, studying this in humans requires large sample sizes and a multi-omics approach [43].

## Conclusion

The gut microbiota’s relationship with the host is complex. It acts as a barrier, signals from the gut to the body, and is linked to androgen production, metabolism, and spermatogenesis. Animal studies support a connection between gut microbiota and tests. Overall, the gut–testis axis offers a promising framework for understanding this relationship and its effects on male reproductive health and well-being.
